# Hay Fever

**Published:** 1911-10

**Authors:** H. S. Persons


### HAY FEVER.

By H. S. Persons, M. D.

Synonyms—The most common synonyms of hay fever are probably hyperesthetic rhinitis, vaso-motor rhinitis, rose cold, and estival catarrh. There have been many others given.

Definition—It is a disease characterized by an acute coryza, coming on suddenly and at stated periods usually, running a certain course and terminating usually as suddenly as its advent—generally with the first frost. There are two kinds—Spring and Autumn.

History—John Bostock first brought this disease into prominence in 1819, although it had been mmentioned by writers as early as the 16th century. The first complete study of it was made by Phoebus in 1854 who issued circulars to the different medical societies then existant. From the reports he received he issued in 1862 a monograph containing a study of 154 cases. He estimated that about 300 cases had been under observation at that time. A little later Helmholtz, in a letter to Binz, gave a detailed description of his own sufferings with it and advanced the theory that it was caused by certain vegetable spores which he found in the discharge from his nose. As stated by Bosworth, it is noteworthy that practically all of the early writers based all of their theories os to its origin on the study of the disease on themselves.

Perhaps the most famous of all students of hay fever was Dr. Blackly, who issued his classical monograph in 1873. As so very little authentic knowledge has been discovered concerning this disease since them, I will briefly detail hs methdos and results.

Blackly was a victim of hay fever and studied the disease in himself. His attacks always began about the 10th of June. He arranged a series of experiments to determine the amount of pollen in the atmosphere at different times and extended his examination over a period of 12 years. The apparatus he devised consisted of a disc of glass, ⅞ of an inch square, mounted on a central staff, which was surmounted by a weather-vane, the disc itself being protected by a hood. This apparatus was exposed under varying atmospheric conditions, and in different situations, usually in an open field. The device for collecting the pollen consisted in placing vertically in a slat, on the surface of this glass plate a microscope slide, one centremetre in diameter, which was coated with a preparation of glycerine, by which the pollen floating in the atmosphere settling on the slide remained adherent to it. This plate was generally exposed day after day for a certain fixeed period, usually about 24 hours, the object of the vane being to keep the vertical slide directed constantly to windward. From the 30th of May to the 7th of June, pollen was found in the air in small quantities. On the 30th of May the number found on the slide was 25, and with this number he began to suffer with perceptible, but not troubblesome swmptoms. On the 8th of June the number rose to 76. On the 11th of June, however, the number of pollen grains found on the small disc rose to a little over 280, and it was at this date that the experimenter began to have unmistakable signs of the commencement of his summer attack in troublesome form. From the 11th of June onward, the amount of pollen varied in a notable degree, until on the 28th of June he counted 880 on his disc. He noticed that on rainy days the amount decreased in a very marked degree, while at the same time his symptoms very notably abated, although on warm days following rain, the number increased very markedly. Moreover, he noticed that a few hours' rai nmade no perceptible difference in his symptoms, whereas, a rain of 24 hours or longer gave very striking relief, in connection with notable decrease in the number of grains found. From the 28th of June onward, there was a gradual diminution, until the 1st of August when the pollen disappeared, with complete abatement of all his symptoms, (Bosworth.)

In 1876 Wyman made a geographical study of the disease, establishing the fact that certain sections of America are exempt. However, some portions which he found free of the disease at that time have since had cases. About this time Beard published a theory that the disease was a neurosis, while Daly combatted this by denying that it was a pure neurosis but was the result of pathological conditions of the naso-pharynx, such as obstructions. Since then various papers have been issued by different authorities, some upholding one theory and some another. Apparently the subject is no nearer a settlement now than 25 years ago.

Etiology—The causes of hay fever may be divided into exciting and predisposing.

Exciting causes—From almost the beginning of our knowledge of hay fever the generally accepted exciting causes by nearly all authorities has been the pollen of certain plants, notably the grammaciae, ambrosia artemisiaefolia (rag-weed) and the solidago virga aurea (golden red.) From these causes has arisen the name "hay-fever." This variety occurs in the late summer. The variety occurring earlier, May or June, is known commonly as

"rose-cold." There have been cases reported, moreover, which have been due to the eminations of certain animals, as cattle, horses, cats, etc.

Predisposing causes—Heredity plays an important part in the predisposition to this disease undoubtedly. Baird reports it as high as 33% in the cases under his observation, and Wyman gives it as 20% in his. All others observers have noticed a large percent in their practice. In my own practice I have three cases occurring among relatives which I will report more fully later.

Many cases are undoubtedly due to neuroses of the naso-pharynx, as deflected septums, enlarged turbinals, neoplasms, possibly adenoids, disease of the accessory sinuses (Ballenger,) and functional derangement of the nervous system (J. N. Mackensie.)

Uric acid has been frequently given as a predisposing cause, but whether this acts directly by making the body fluids acid, or by a general derangement of the chemical functions, has not been settled.

Age plays some part in this disease. The most prolific age is about twenty years, though cases occur among young babies and I have one case which came on after sixty years of age.

Sex— Males are more susceptible than females, though in my opinion, in these cases where neuroses are the causative factor the reverse is the case.

Race also has a bearing. Angle-Saxons, Germans and French are the most susceptible. I have seen one case among negroes, a mulatto.

Location plays a very important part in its causation. It is more common in Northern than Southern climates; low, flat countries (particularly those bordering on large bodies of waret) than high, dry regions. Until recently it was rarely seen west of the Mississippi river, and the region of the White mountains, the Adironkacks, the Catskills were supposed to be entirely free of it, but now cases are found even there.

Social conditions—This is called the disease of the rich for it is rarely found among the very poor—which may account for its scarcity among negroes.

Pathology—The pathological changes, in appearance, vary very little from the condition observed in an attack of acute rhinitis. Ordinarily the nose will secrete from twelve to sixteen ounces of watery serum per day the amount varying to meet the different atmospheric condition. In this disease there is a very great increase in the watery serum, which at the same time becomes glairy and acrid. It is generally supposed that the pollen, acting on the nerve terminals, causes a vaso-motor paralysis, with a dilatation of the venous sinuses, which permits of a freer escape of the serum. The veins, or sinuses, spoken of here, are those composing the turbinal bodies and not those of the mucous membrane lining the interior of the nose. The predisposing neurosis probably weakens the vast-motor control which the sympathetic and trigeminus nerves exercise over the sinuses, making them very susceptible to the action of pollen (Bosworth.)

Symptomatology—The onset is usually sudden the attack in this region commencing on the 28th of August, as a rule. The first symptoms usually are a feeling of malaise, a slight elevation of temperature, together with attacks of sneezing. Then the nose closes, the eyes become red and there follows headache more or less severe. About this time itching becomes very severe. It is located either in the roof of the mouth near the soft palate, in the nose, or both places and frequently the eyes itch and burn. With the congestion and stoppage of the nose the watery secretion increases in amount, becomes glairy, very acrid (so much so that the interior nares and upper lip is soon excoriated) and later contains mucus, and maybe a little pus. During sleep there may be a little improvement in the nasal condition. The patient usually complains of dryness and tickling of the throat, possibly tinnitus aurium. In nearly all cases the appetite is markedly impaired and the patient's general condition suffers as a result. An examination of the nose shows the turbinals much congested and the nares closed. This congestion may extend to the soft palate and pharynx. The glairy serum is so free that in making an application to the membrane the medicine is frequently washed out wtihout having any effect. In fact, in three cases which I will mention later, the applicaion of any of the supra-renal extracts had no effects unless I first made an application of a cocaine solution. The symptoms given above are not continuous by any means, but they are usually so severe that a patient is frequently prevented from performing his usual labors.

Geographical distribution—It was formerly thought, as set forth by Beard in his treatise on Autumnal Catarrh, that hay fever was limited in its distribution to a portion of the U. S. east of the Mississippi river and lying between the 35th and 45th parallels of latitude. Not all of this territory had it, however, as Canada, the Adirondacks, the elevated through the center of New York state and the Appalachian range were exempt. Also all of the Southern States, except small areas around Milledgeville, Ga., Montgomery, Ala. and Beauford, S. C. were exempt. Later investigations, however, have proven this not true. There is now no portion of the U. S. considered completely exempt, not even the White Mountains. Patients who have the disease will be relieved in nearly all cases when they go to the White Mountains in spite of the occasional existence of cases there.

Course and duration—As I have said before, most cases in this climate commence on August 28th, or a few days later. I have three old cases, who will let no operative work be done in an effort to cure them, who come to my office regularly on August 29th, the disease having commenced on the previous day. This date varies in different sections of the country and in different countries. The kind known as "rose-cold" usually comes on in June or possibly May. I have referred previously to three cases which I will take up here, as they are the only unusual cases I have had. Each of these cases have started in January, which is an unusual time for them to commence, but as they showed every symptom of hay fever I have not hesitated to so classify them. They were a sister, about 22 years of age, brother about 16 years of age,and a female, a cousin, 14 years old. The sister came to me first in January, 1909. I treated her for temporary relief until the disease ceased about the middle of March and later I removed portions of the inferior turbinals and cauterized the sensitive areas as I found them. The next two years she was exempt, althouhg she gave a history of having had the same symptoms at least two years before coming to me. The brother and the cousin both came in January, 1910, with the same symptoms as the preceding case, ran the same course, and since then have had the same treatment. They have remained well since then. Another peculiar feature of all of these cases was that each seems to have an idiosyncrasy for the supra-renal extracts. Every time I would try to use them the congestion would be worse, the discharge increased in amount and the sneezing made much worse. On the other hand, a 4% solution of cocaine would work like a charm and after I had gotten a good result with it I could then use a supra-renal extract and increase the result. Each of these cases, from all of the symptoms, were neuroses, I am sure.

Diagnosis—About the only disease which which hay fever can be confounded is acute rhinitis, and many cases of the former are doubtless diagnosed as the latter. In acute rhinitis there is no periodicity. While there is more or less congestion (according to the amount of the disease a person has) it is reddish and the time of the serous discharge is short, quickly becoming mucus, or muco-purulent. Also the paroxysms of sneezing are never so severe as in hay fever, nor do they extend over the length of time of the serous discharge is short quickly becoming mucous, previous attacks (unless you happen to see a patient with the first attack); there is the sudden onset; there is a marked congestion of the turbinals, showing bluish-gray instead of bright red; there is the viscid serum during the entire attack, which frequently looks stringy; and, finally, the paroxysms of sneezing are very severe. Also the congestion and itching of the eyes, with their photophobia and lachrimation. In fact, there should be no chance of a mistake, certainly after two or three visits.

Prognosis—Hay fever is never a fatal disease so we are only concerned with its cure or relief. Bosworth gives a record of 121 cases in his private practice, and as his results give about the average of all cases which I have found in the literature at my disposal, I shall quote them alone. The 121 cases resulted as follows:

| | |
|---|---|
| Cured | 51 |
| Relieved | 43 |
| Unreleived | 13 |
| Results unknown | 14 |

From the above it will be seen that we are safe in promising a favorable result in a large maiojrijtjy of cases, though it would be unwise to promise a sure in any particular case. It does not seem to make any difference in results as between a recent case and one of long standing, as we are just as apt to be successful in one as the other. The form known as "hay-asthma" can be treated as successfully as the straight hay fever, though past experience has probably shown "rose-cold" to be more amenable to treatment than any form. Young people can be more successfully treated than old. I have records of only eight cases and they have been within the past six years. Before that I kept no records. Three of these I have mentioned in detail. Of the other five, all of which were regular hay fever types, three would permit no operative treatment at all, so all I have done was for tempory relief. Of the other two, one was treated six years ago and was a complete cure. The other was treated in 1908 and while the cure has not been complete, the relief has been so great that the patient has no trouble in continuing his daily occupation. The symptoms in the last two years have disappeared long before the first frost.

Treatment—This is divided into prophylactic, general and local. The prophylactic treatment should commence from two to three months previous to the attack. We should, so far as possibly, correct any errors of diet; should correct constipation; cause a patient who leads a sedentary life to take more outdoor exercise; should advise cold baths, possibly salt baths are better; and should curtail, or cut off, alcoholic liquors. More important, however, is to correct any pathological condition existing in the nose. Deflections should be corrected, hypertrophies reduced, polypi removed and any disease of the accessory sinuses treated. Ballenger believes that many cases of hay fever are caused by disease of these sinuses, and cites several cases which he cured by removing this predisposing cause. N. J. Wilson believes that in these cases the urine is always charged with uric and pays special attention to its correction. It is not always easy to select the particular medicine which suits each individual case, but he gives sodium salicylate or quinine hydrochlorate in large doses usually.

The local treatment of hay fever varies, depending on the physician giving it. Personally, I think it best to cleanse the nose thoroughly with an alkaline spray and then reduce the congestion by the application of some of the supra-renal extracts. In some cases, where these extracts do not have the desired effect, I use a 4% solution of cocaine on cotton, but as I have seen one case of cocaine habit formed during an attack of this disease, I never tell my patient what I have been using, nor do I give a prescription containing cocaine. After the congestion is reduced, if I can locate any particular spots which cause sneezing, I use the electric cautery to smear them over, using the flat side of the blade. In no case do I do any operative work during an attack. In all cases I give an alkaline spray containing one of the supra-venal extracts for the patients' use at home. When I find it beneficial I also give an oily spray containing menthol and other drugs, believing that this will make the patient more comfortable, and help to keep the nasal passages open longer. During last year I tried wool medicated with menthol placed in the anterior part of the nose, and one patient reported that it made his condition slightly more comfortable, while the other one could see no benefit. I tried pollantin in three cases—the two just referred to and one other which I subsequently cured, and got no results at all that I could see. I do not use it at all now.

The eye symptoms can be relieved by the same alkaline spray containing the supra-renal extract that is used in the nose, though I make it weaker.

In conclusion I wish to repeat that hay fever patients should never be discouraged from taking treatment, but, on the other hand, shoud bel directed to some specialist, as the majority can either be cured or benefitted. Any curtailment of the distressing symptoms is a god-send to the sufferer. It is always best to send them from six weeks to two months before the commencement of the attack.

---

### THE SOUTHERN MEDICAL ASSOCIATION.

New Oreans, La., Sept. 15, 1911.

*To the Members of the Medical Profession of the South*:

The Southern Medical Association needs all of the profession in the South on its roll of membership, and it would be gratifying to the adminstration of the present year if those who may be nterested and who are not already members would join before the Hattiesburg meeting, November 14, 15 and 16.

The purposes of the association are wholly those which govern us, and the opportunit of ygathering annually in our own section to further our own interests should appeal to us. Each of the meetings has been successful, enjoyable and enlightening, and the coming meeting promises more. All of the sections are busy preparing their programmes, and the profession in Hattiesburg is ready to welcome their confreres with open arms.

Our genial and industrious Secretary, Dr. Harris, is anxious to enoll a large increase in membership, and to him you may send application.

There are many matters of mutual interest which will come up this year ,and the spirit of these meetings is full of a growing concern for the future of our public health in the South as well as the preseration of our tradvitions.

Will each of you who may read this letter plan to come to Hattiesburg in November and join the movement for a common good?

Yours fraternally and faithfully,

ISADORE DYER, *President*.

